# Supplementary material for: Teaching fish new tricks: Repeated exposure to a velocity barrier improves passage performance
Source: PLoS One. 2025 Aug 8;20(8):e0329371. doi: 10.1371/journal.pone.0329371 (PMC12334001; doi:10.1371/journal.pone.0329371)
Supplement: Attachment — Submitted filename: renamed_3f713.pdf [file pone.0329371.s003.pdf]

20250323

PONE-D-24-53465

L105__

Were the juveniles caught before they had completed their upstream migration to their habitat? Or was the capture location their habitat?

This may be considered one of the reasons why "fish failed to enter the raceway each day."

L117__

Did they reuse the waterway described in (39), or did they use the features of (39)?

I would like you to add a little more explanation about the introduction of the waterway so that it can be understood without reading (39).

If PVC is an abbreviation for something, I would like an explanation for that.

L130__

If the subject of the paper is "velocity barrier," I think there should be some experiments or discussion on "rush speed" as well as "critical swimming speed."

L134__

It would be good if the design concept of the "40°　angle" was described.

I would like to see the concept of the water flow not starting from a single point in the center, but evenly flowing from a 0.5m width or from multiple outlets described.

L137__

It is suggested that the "starting pool" refers to the space between (D) and (E) on the right, but a more specific description would be needed.

If there was an elevation drawing, it would be easier to understand the following points:

- "with a consistent water depth of 0.2 m maintained throughout all trials."
- "Measurements were taken at three heights: 20%, 60%, and 80% of the water column above the raceway floor."
- The height at which the starting water flow occurs

Does "successful "passage" of the fishway" mean the passage of the entire fish body?

L165__

Please state in the caption of the image that the water depth is 50 cm or give a specific water depth.

L180__

I would like to know if "Fish weight and condition factor were not included in any of the statistical models due to high correlation with length (0.85 and 0.76 respectively)." is a general statement or a result calculated this time.

L182__

Does "in the downstream pool" refer to the "starting pool"?

L212__

Regarding "the end of the 30 min trial," I was unable to find an explanation for why the maximum trial time was set at 30 minutes.

Please describe how the juvenile fish were managed between experiments (23 hours?).

L220__

It seems to me that this equation only applies to fish where the relationship between body length and weight is constant, such as "young *Plecoglossus altivelis*" or "young *Oncorhynchus masou*" but will this be considered?

L251__

If there were a clear explanation somewhere that larger fish are easier to approach, this statement would be easier to understand, and it could also be used in discussions about small fish.

L260__

Although Figure 3 appears before Table 2 in the text, why are they listed in the opposite order?

L264__

Considering the development of the discussion in this study, if the "effect of day" could be rephrased somewhere with the intention of "effect of frequency," mutual understanding would advance.

L265__

Considering this case in which the fish's body length has a significant effect, it would be helpful to clarify the relationship between the fish's body length and their charging speed.

L268__

Does "this increase appeared to asymptote and even decrease after Day 4," indicate that while repeated attempts increase the probability of success, repeated attempts also cause some individuals to give up on the upstream migration?

L276__

I would like to know which values in Table 2 are used and how the calculation results in "HR = 1.0."

I did not understand how Figure 4 should be interpreted to support this statement.

L279__

Although Figure 4 appears before Table 3 in the text, why are they presented in the opposite order?

L314__

I felt that this was not an argument about attempting to overcome a single barrier multiple times, but rather about the need to overcome multiple barriers to reach a habitat.

Because this is an important argument, I feel it would be better to make this point of view the purpose of the paper and make it a bit clearer.
