# Supplementary material for: Teaching fish new tricks: Repeated exposure to a velocity barrier improves passage performance
Source: PLoS One. 2025 Aug 8;20(8):e0329371. doi: 10.1371/journal.pone.0329371 (PMC12334001; doi:10.1371/journal.pone.0329371)
Supplement: Attachment — Submitted filename: Review report.docx [file pone.0329371.s001.docx]

**Comments on the manuscript**

Thank you for the opportunity to review this manuscript. The paper is well-written and structured, and it addresses an important topic: how repeated exposure to a high-velocity water barrier affects the ability of juvenile *Galaxias maculatus* to pass through it. The experiment involved subjecting individual fish to the same high-speed water conditions in a raceway over five consecutive days. They found that the proportion of fish successfully passing the barrier increased significantly from 40% on Day 1 to 63% on Day 5. Time-to-event analysis revealed that fish passed the barrier more quickly by Day 5 than Day 1 but did not significantly improve approach or entry rates into the raceway. The study suggests that cognition and spatial memory play a role in improving passage performance through velocity barriers, but other factors, such as attraction flows, are also important. However, there are a few points that could be strengthened:

1. A total of 40 fish were used in the experiment. Although this sample size may be adequate, a larger sample size could provide more robust results and increase the statistical power of the study.

2. The study focuses on one species (*Galaxias maculatus*), which may not fully represent cognitive responses in other fish species.

3. While the study discusses cognition and memory, it does not explicitly assess fish personality traits (e.g., boldness or exploratory behavior), which could influence passage success.

4. While water temperature was monitored, other environmental factors, such as dissolved oxygen and pH levels, were not explicitly addressed. Fluctuations in these factors could potentially influence fish behavior and passage performance.

5. The experimental raceway does not fully replicate natural stream conditions, which may affect generalizability.

6. The study does not assess whether repeated exposure induces stress or fatigue, which could influence fish behavior.

Therefore, based on the concerns outlined above, I recommend that this manuscript be accepted for publication after major revision.
