# Supplementary material for: Teaching fish new tricks: Repeated exposure to a velocity barrier improves passage performance
Source: PLoS One. 2025 Aug 8;20(8):e0329371. doi: 10.1371/journal.pone.0329371 (PMC12334001; doi:10.1371/journal.pone.0329371)
Supplement: Attachment — Submitted filename: Response_to_reviewers.docx [file pone.0329371.s004.docx]

Manuscript ID: PONE-D-24-53465

Dear Editor Dr Mizanur Rahman,

Thank you for the opportunity to revise our manuscript “Teaching fish new tricks: Repeated exposure to a velocity barrier improves passage performance ” (Manuscript ID PONE-D-24-53465) for consideration for publication in PLOS ONE. We appreciate the constructive comments provided by the two peer reviewers. We hope that our revision has suitably addressed all identified concerns.

Please see below for a point-by-point summary of changes as raised by reviewers (in RED). We have also provided both a tracked change and clean copy of the revised manuscript for your consideration. Any references to line numbers in our response to the reviewers are based on the tracked change version.

On behalf of the full authorship,

Yours sincerely,

Rachel Crawford

National Institute of Water and Atmospheric Research, Hamilton, New Zealand

rachelmbcrawford@gmail.com

**Reviewer 1**:

Thank you for the opportunity to review this manuscript. The paper is well-written and structured, and it addresses an important topic: how repeated exposure to a high-velocity water barrier affects the ability of juvenile Galaxias maculatus to pass through it. The experiment involved subjecting individual fish to the same high-speed water conditions in a raceway over five consecutive days. They found that the proportion of fish successfully passing the barrier increased significantly from 40% on Day 1 to 63% on Day 5. Time-to-event analysis revealed that fish passed the barrier more quickly by Day 5 than Day 1 but did not significantly improve approach or entry rates into the raceway. The study suggests that cognition and spatial memory play a role in improving passage performance through velocity barriers, but other factors, such as attraction flows, are also important. However, there are a few points that could be strengthened:

Thank you for your feedback. We have made revisions in response to your comments as described below.

1. A total of 40 fish were used in the experiment. Although this sample size may be adequate, a larger sample size could provide more robust results and increase the statistical power of the study.

While a larger sample size would produce more robust results, we were limited logistically, in terms of seasonal availability and amount of time it takes to conduct trials, which limited the number of fish we could use in this study.

2. The study focuses on one species (Galaxias maculatus), which may not fully represent cognitive responses in other fish species.

This is correct that *Galaxias maculatus* may not fully represent cognitive responses in other fishes. We hope to use this as a foundation for future work to look at cognitive abilities across more species in the future. We have also added in some discussion around this limitation (Lines 413-415).

3. While the study discusses cognition and memory, it does not explicitly assess fish personality traits (e.g., boldness or exploratory behavior), which could influence passage success.

In hindsight, individual fish personality is something we wish we had assessed. We have included some theories on how fish personality may have influenced passage success in our experimental raceway rather than just cognitive ability alone (Lines 443-453).

4. While water temperature was monitored, other environmental factors, such as dissolved oxygen and pH levels, were not explicitly addressed. Fluctuations in these factors could potentially influence fish behavior and passage performance.

While other environmental variables were not monitored, such as dissolved oxygen and pH levels, we expected the variability in these variables to be relatively minimal. The flume was open to the air and had highly turbulent flow (Reynolds number = 220, 588). Any differences in dissolved oxygen would be minimal and strongly correlated with temperature. For pH, the fish were very small relative to the volume of the experimental setup, and no other factors were present that would influence pH, as the system used raw, dechlorinated water. See lines 143-148.

5. The experimental raceway does not fully replicate natural stream conditions, which may affect generalizability.

This limitation has been discussed in the Discussion section (Lines 407-413). However, the controlled environment of the laboratory flume offers several advantages, including the ability to control for confounding factors. This allows us to more accurately isolate learning effects from other influences, such as variation in group size, as well as ensuring consistent flow conditions and water speeds across all trials. In the first instance we need to have a controlled experimental setup which can then inform field tests in the future.

6. The study does not assess whether repeated exposure induces stress or fatigue, which could influence fish behavior.

This is a valid point—measuring blood chemistry as a proxy for stress would be a valuable addition in future experiments.

Therefore, based on the concerns outlined above, I recommend that this manuscript be accepted for publication after major revision.

It is not feasible to repeat these experiments with a greater number of individuals or with additional species at this stage. If the editor considers these additions essential for publication in this journal, we are prepared to withdraw the manuscript. While we acknowledge that including additional species or increasing the sample size would strengthen the study, we believe the current work offers a valuable contribution to the literature and provides a solid foundation for future research.

**Reviewer 2:**

The study addresses an important ecological issue: the impact of anthropogenic barriers on fish migration. The findings have implications for conservation and management of freshwater ecosystems, particularly for small-bodied, non-salmonid species. The focus on *Galaxias maculatus*, an obligate migratory species, adds ecological relevance, as this species is widely distributed in the Southern Hemisphere and faces challenges from human-altered environments. Following points need to be addressed for improvement in manuscript.

Thank you for your suggestions and recommendations for the improvement of our manuscript.

1. At line 109, Elaborate reason why authors kept fish in 6 ppt salinity. Does it match the source of procurement? Give reason.

The use of 6 ppt salinity in quarantine tanks is to help prevent disease and kill any potential disease the fish may bring with them from the river. This is explained at line 125.

2. Expand the sample size and replicate the experiment across multiple groups or populations to improve the robustness of the findings.

While a larger sample size would produce more robust results, we were limited logistically, in terms of seasonal availability and amount of time it takes to conduct trials, which limited the number of fish we could use in this study.

3. Consider reusing individual fish in follow-up experiments to assess individual variability in learning and performance.

In hindsight, this would be a good suggestion, however, it was not feasible given the seasonality and timing constraints of this experiment. As suggested to Reviewer 1, in the future we would like to assess individual fish personality and take that into account in our experiments.

4. Isn't it better to consider reusing individual fish in follow-up experiments to assess individual variability in learning and performance.

This appears to make the same point as item 3, and as noted it was unfortunately not feasible to conduct further trials in the current study.

5. Provide specific recommendations for conservation and management based on the findings, such as how to improve fishway design or mitigate the impacts of barriers on fish migration.

We have added some additional discussion around this topic, please see lines 421-430.

6. The experimental setup, while controlled, may not fully replicate real-world conditions. For example, the raceway's simplified structure and uniform flow conditions do not account for the sample size (40 fish) is relatively small, and the study does not mention whether the experiment was replicated across multiple groups or populations. Replication would strengthen the robustness of the findings.

Similar to Reviewer 1’s comments, while a larger sample size would produce more robust results, we were limited logistically, in terms of seasonal availability and amount of time it takes to conduct trials, which limited the number of fish we could use in this study. Additionally, while only juvenile *Galaxias maculatus* were used in this study, care should be taken not to over-extrapolate findings to other populations or other fish species (Lines 413-415). We have discussed limitations to this study in the discussion section, lines 407-415.

7. Consider conducting follow-up experiments in more complex environments or with different types of barriers to assess the generalizability of the findings. Incorporate variability in flow conditions or barrier design to better mimic real-world scenarios. Include direct measures of spatial learning, memory, and behavioral traits (e.g., boldness, exploration) to strengthen the interpretation of results.

As suggested to Reviewer 1, this limitation has been discussed in the Discussion section (Lines 443-453). However, the controlled environment of the laboratory flume offers several advantages, including the ability to control for confounding factors. This allows us to more accurately isolate learning effects from other influences, such as variation in group size, as well as ensuring consistent flow conditions and water speeds across all trials. In the first instance we need to have a controlled experimental setup which can then inform field tests in the future.

The manuscript presents a well-designed and executed study with important implications for understanding fish passage through anthropogenic barriers. However, the ecological realism, exploration of behavioral mechanisms, and statistical reporting could be improved. Addressing these weaknesses would strengthen the manuscript and enhance its contribution to the field of freshwater ecology and conservation.

In lieu of any specific statistical comments from this reviewer, we are using Reviewer 3’s comments and have made changes in line with their recommendations.

**Reviewer 3:**

The manuscript presents a technically sound scientific investigation, supported by a detailed and rigorous methodology. It includes all the essential elements of a well-structured study, such as the description of the required permits and approvals for fish collection, experimental trials, setup and experimental protocol, as well as statistical analysis.

The results are presented clearly and in alignment with the methodology, allowing the reader to assess whether the objectives were achieved. Additionally, the analyses are well-founded, providing a robust interpretation of the data while transparently acknowledging the study’s limitations. In particular, the article highlights the ability of fish to improve their passage success with repeated exposures but also recognizes that it remains uncertain whether this learning is transferable to new obstacles.

A valuable aspect of the manuscript is that, in addition to addressing its research question, it provides relevant findings for the management of fish passage structures. It highlights challenges in fish attraction and entry, as well as the potential effect of shyness in some individuals, suggesting that management strategies should focus not only on the physical structure of passageways but also on improving fish attraction.
Overall, this study significantly contributes to the understanding of fish passage efficiency and provides valuable information for both science and the management of aquatic ecosystems.
Thank you very much for your feedback and your suggestions.
Editorial Recommendations
Some structural adjustments are necessary in the manuscript:
**a)** In the Modelling Binary Success section, when mentioning p-values, the text should correctly direct the reader to Table 2.

Thank you for pointing this out, this has now been corrected.
**b)** In line 278, "Table 2" should be changed to "Table 4."

Thank you for pointing this out, this has now been corrected.
**c)** It is recommended to reorganize the order of the paragraphs to maintain the logical sequence of the results, placing Table 3 before Table 4.

Thank you for pointing this out, this has now been corrected.

**2.** I consider that the statistical analysis in the manuscript has been conducted appropriately and rigorously. The authors applied a solid approach using generalized binomial generalized linear mixed models (GLMM) to model binary success in approach, entry, and successful passage, incorporating random effects to capture individual variability in fish. Additionally, they employed Cox regression to assess event rates (approach rate, entry rate, and passage rate), allowing for the interpretation of the effects of repeated exposure over time and generating Hazard Ratios (HR) to measure the instantaneous probability of success at each threshold of the experimental channel.

However, I found an inconsistency in the application of the Hosmer-Lemeshow goodness-of-fit test. In the methodology, it is stated that this test was used to evaluate model fit in the GLMM for all three events (approach, entry, and successful passage), but in the results, its use is only reported for the entry model. This raises questions about whether it was also applied to the other two events or if its reporting was omitted.
Furthermore, I consider that the Hosmer-Lemeshow test is not the most appropriate for evaluating the fit of GLMM models, as it does not account for the random effects characteristic of these models. I suggest using more suitable methods, such as residual inspection, AIC/BIC comparison, or the simulation of predicted versus observed values. If a new model fit method is adopted, I recommend applying it uniformly across all events and including the corresponding results in the results section to ensure consistency with what is described in the methodology.

Despite this observation, I believe that the analyses used in the study are appropriate for addressing the research question and allow for well-supported conclusions.

Thank you for pointing this out. After doing more research, we found that you are correct and the AIC comparison is more appropriate for GLMM models with random effects. Using the AIC comparison, the GLMM model chosen for each threshold meets the AIC selection criteria (Lines 247-251).
**3.** In the manuscript, the authors indicate that the data will be available through the Zenodo digital repository and provide the corresponding doi: 10.5281/zenodo.14194951. However, I was unable to access this DOI or find an active link.
I suggest that the authors verify the availability of the link and confirm that the data are correctly deposited in the repository to ensure open access and transparency in the publication.

The data are now available via the Zenodo digital repository: https://doi.org/10.5281/zenodo.14194951

**4**. The manuscript is written clearly, without ambiguities, and in a comprehensible level of English. During my review, I did not identify any typographical or grammatical errors that would affect the understanding of the content. However, since English is not my native language, my assessment of full compliance with linguistic standards is limited.

**Reviewer 4:**

Were the juveniles caught before they had completed their upstream migration to their habitat? Or was the capture location their habitat?
This may be considered one of the reasons why "fish failed to enter the raceway each day."
Juvenile fish were captured at the beginning of their upstream migration to control for migratory urge across individuals (Lines 120-122).
L117＿
Did they reuse the waterway described in (39), or did they use the features of (39)?
I would like you to add a little more explanation about the introduction of the waterway so that it can be understood without reading (39).

We have clarified in text that this study used the same flume and same setup as described in (39).

If PVC is an abbreviation for something, I would like an explanation for that.

PVC stands for polyvinyl chloride. We have added this abbreviation to the paper.

L130＿
If the subject of the paper is "velocity barrier," I think there should be some experiments or discussion on "rush speed" as well as "critical swimming speed."
Thank you for pointing this out, as burst swimming (“rush speed” as you describe it) is most likely what the fish will use to pass the velocity barrier. We have now added in some discussion of this (Lines 153-159).
L134＿
It would be good if the design concept of the "40° angle" was described.
I would like to see the concept of the water flow not starting from a single point in the center, but evenly flowing from a 0.5m width or from multiple outlets described.
The 40° was chosen to provide a relatively smooth transition into and out of the raceway, allowing for a smoother acceleration of flow without introducing turbulence of a right angle. However, the specific angle was not chosen for a particular reason other than to minimise turbulence and fit in with the spatial constraints of the flume. The water flowed evenly across the 0.5m width of the flume, not from a single point. See lines 163-165.
L137＿
It is suggested that the "starting pool" refers to the space between (D) and (E) on the right, but a more specific description would be needed.
If there was an elevation drawing, it would be easier to understand the following points:
- "with a consistent water depth of 0.2 m maintained throughout all trials."
- "Measurements were taken at three heights: 20%, 60%, and 80% of the water column above the raceway floor."
- The height at which the starting water flow occurs
Does "successful "passage" of the fishway" mean the passage of the entire fish body?

We have added further clarification in both the main text and the figure caption (Lines 169-165, 184-185). We have changed the terminology to “starting section” to indicate there is no change in bed elevation in this section of the flume. There was no change in bed elevation across the flume—rather, the setup included an unobstructed section leading into the narrowing of the raceway. Water depth was consistently 0.2 m throughout all trials. ADV measurements were taken based on standard protocol at heights of 20%, 60%, and 80% of the water column, corresponding to 0.04 m, 0.12 m, and 0.16 m above the raceway floor, respectively. We believe that an additional cross-sectional figure would not provide any further useful information.

L165＿
Please state in the caption of the image that the water depth is 50 cm or give a specific water depth.

We have added in the figure caption that the water depth was 0.2 m across the whole flume (Lines 173-175).
L180＿
I would like to know if "Fish weight and condition factor were not included in any of the statistical models due to high correlation with length (0.85 and 0.76 respectively)." is a general statement or a result calculated this time.

The condition factor for each fish was calculated using Fulton's equation, and correlations between fish weight, condition factor, and length were assessed in RStudio prior to running the models (Lines 217-218)
L182＿
Does "in the downstream pool" refer to the "starting pool"?

That is correct. We have changed the wording to “starting section” throughout to be more consistent and remove confusion around bed elevation (line 219).

L212＿
Regarding "the end of the 30 min trial," I was unable to find an explanation for why the maximum trial time was set at 30 minutes.
Please describe how the juvenile fish were managed between experiments (23 hours?).
We have added some text in the methods section as to why a 30 min maximum trial time was chosen (Lines 230-235). Trial duration was set to a maximum of 30 minutes based on preliminary observations indicating that fish which had not successfully passed through the raceway within this period were unlikely to do so even after several additional hours. This duration also allowed for the practical scheduling of multiple trials per day. After each trial, fish were returned to the holding tank and allowed to recover until the subsequent trial, conducted 24 hours later.
L220＿
It seems to me that this equation only applies to fish where the relationship between body length and weight is constant, such as "young Plecoglossus altivelis" or "young Oncorhynchus masou" but will this be considered?

These time-to-event (or survival analysis) equations has been used widely across both medical science as well as fish ecology, and we believe it is well suited to our statistical needs in the manuscript.
L251＿
If there were a clear explanation somewhere that larger fish are easier to approach, this statement would be easier to understand, and it could also be used in discussions about small fish.
We have added in more detail that larger fish are more likely to approach the raceway than smaller fish (Lines 296-297).
L260＿
Although Figure 3 appears before Table 2 in the text, why are they listed in the opposite order?

Thank you for pointing this out we have now reordered the Tables and Figures.
L264＿
Considering the development of the discussion in this study, if the "effect of day" could be rephrased somewhere with the intention of "effect of frequency," mutual understanding would advance.

We have now changed this to “effect of repeated exposure” to enhance clarity throughout.
L265＿
Considering this case in which the fish's body length has a significant effect, it would be helpful to clarify the relationship between the fish's body length and their charging speed.

We have added in text to explain that larger fish are more likely to be successful than smaller fish.
L268＿
Does "this increase appeared to asymptote and even decrease after Day 4," indicate that while repeated attempts increase the probability of success, repeated attempts also cause some individuals to give up on the upstream migration?
The asymptote may indicate that there are other factors at play other than just improved cognition. As you point out, some fish may “learn” that they don’t want to swim through the raceway, or there may be other factors, such as personality, that prevent them from being successful.
L276＿
I would like to know which values in Table 2 are used and how the calculation results in "HR = 1.0."
I did not understand how Figure 4 should be interpreted to support this statement.

We realize that Table 2 was incorrectly referenced here and should be referencing Table 3. We have also added in a sentence to the figure caption to describe that when the lines diverge, this indicates a hazard ratio that differs from 1. Where the lines overlap, it suggests a hazard ratio of 1, indicating no significant difference between exposure days
L279＿
Although Figure 4 appears before Table 3 in the text, why are they presented in the opposite order?

We have reordered the Figure and Table in the text.
L314＿
I felt that this was not an argument about attempting to overcome a single barrier multiple times, but rather about the need to overcome multiple barriers to reach a habitat.
Because this is an important argument, I feel it would be better to make this point of view the purpose of the paper and make it a bit clearer.

We have added in additional text to highlight that this study repeatedly exposed fish to the same barrier as a proxy for the cumulative effects of encountering multiple barriers along a migration route (Lines 382-384).
