# Supplementary material for: Teaching fish new tricks: Repeated exposure to a velocity barrier improves passage performance
Source: PLoS One. 2025 Aug 8;20(8):e0329371. doi: 10.1371/journal.pone.0329371 (PMC12334001; doi:10.1371/journal.pone.0329371)
Supplement: Attachment — Submitted filename: Reviewer Comments.docx [file pone.0329371.s002.docx]

The study addresses an important ecological issue: the impact of anthropogenic barriers on fish migration. The findings have implications for conservation and management of freshwater ecosystems, particularly for small-bodied, non-salmonid species. The focus on *Galaxias maculatus*, an obligate migratory species, adds ecological relevance, as this species is widely distributed in the Southern Hemisphere and faces challenges from human-altered environments. Following points needs to be addressed for improvement in manuscript.

Areas of Revision

1. At line 109, Elaborate reason why authors kept fish in 6 ppt salinity. Does it match the source of procurement? Give reason.
2. Expand the sample size and replicate the experiment across multiple groups or populations to improve the robustness of the findings.
3. Consider reusing individual fish in follow-up experiments to assess individual variability in learning and performance.
4. Isn't it better to consider reusing individual fish in follow-up experiments to assess individual variability in learning and performance.
5. Provide specific recommendations for conservation and management based on the findings, such as how to improve fishway design or mitigate the impacts of barriers on fish migration.
6. The experimental setup, while controlled, may not fully replicate real-world conditions. For example, the raceway's simplified structure and uniform flow conditions do not account for the The sample size (40 fish) is relatively small, and the study does not mention whether the experiment was replicated across multiple groups or populations. Replication would strengthen the robustness of the findings.
7. Consider conducting follow-up experiments in more complex environments or with different types of barriers to assess the generalizability of the findings. Incorporate variability in flow conditions or barrier design to better mimic real-world scenarios. Include direct measures of spatial learning, memory, and behavioral traits (e.g., boldness, exploration) to strengthen the interpretation of results.

The manuscript presents a well-designed and executed study with important implications for understanding fish passage through anthropogenic barriers. However, the ecological realism, exploration of behavioral mechanisms, and statistical reporting could be improved. Addressing these weaknesses would strengthen the manuscript and enhance its contribution to the field of freshwater ecology and conservation.
